# Foot posture, foot function and low back pain: the Framingham Foot Study

**DOI:** 10.1186/1757-1146-6-S1-O27

**Published:** 2013-05-31

**Authors:** Hylton B Menz, Alyssa B Dufour, Jody L Riskowski, Howard J Hillstrom, Marian T Hannan

**Affiliations:** 1 Lower Extremity and Gait Studies Program, La Trobe University, Bundoora, Victoria 3086, Australia; 2 Institute for Aging Research, Hebrew SeniorLife, Boston, Massachusetts 02131, USA; 3 Harvard Medical School, Boston, Massachusetts 02131, USA; 4 Boston University School of Public Health, Boston, Massachusetts 02131, USA; 5 Hospital for Special Surgery, New York, New York 10021, USA

## Background

Low back pain is a highly prevalent problem world-wide. Abnormal foot posture and function have been proposed as possible risk factors for low back pain, but this has not been explored in detail.

## Methods

Data were collected on 1,930 members of the Framingham Study who completed the foot examination in 2002–2005. Low back pain, aching or stiffness on most days was documented on a body chart. Foot posture and foot function were evaluated using the Tekscan MatScan^®^ system. Foot posture was categorized as normal, planus or cavus using static weightbearing measurements of the arch index. Foot function was categorized as normal, pronated or supinated using the center of pressure excursion index derived from dynamic foot pressure measurements. Asymmetry in foot posture and foot function was also determined. Sex-specific multivariate logistic regression models were used to examine the associations of foot posture, foot function and asymmetry with low back pain, adjusting for relevant confounding variables.

## Results

Low back pain was reported by 661 (34%) participants, including 404 (37%) women and 257 (30%) men. Foot posture showed no association with low back pain. However, pronated foot function was significantly associated with low back pain in women (odds ratio [OR] = 1.51, 95% confidence interval [CI] 1.1 to 2.07, P=0.011) and this association remained significant after adjusting for age, weight, smoking and depressive symptoms (OR = 1.48, 95% CI 1.07 to 2.05, P=0.018). Asymmetry in foot posture or foot function was not significantly associated with low back pain.

## Conclusion

This is the first population-based study to examine the associations of foot posture and function with low back pain using objective biomechanical measurements. The findings suggest that pronated foot function may contribute to the development of low back symptoms in women. Interventions which modify abnormal foot function, such as foot orthoses, may therefore have a role in the prevention and treatment of low back pain.

